# Robust Broadband Differential Beamforming for Fully Steerable Linear Superarrays with Prescribed Nulls

**DOI:** 10.3390/s26175502

**Published:** 2026-08-30

**Authors:** Yue Wang, Yankai Zhang, Jiafeng Ding, Jingjing Ning, Qiaoxi Zhu

**Affiliations:** 1 Key Laboratory of Functional Materials and Devices for Informatics of Anhui Educational Institutions, Fuyang Normal University, Fuyang 236037, China; 2024221108@stu.fynu.edu.cn (Y.W.); 23221131@stu.fynu.edu.cn (J.D.); jning@fynu.edu.cn (J.N.); 2 School of Physics and Electronic Engineering, Fuyang Normal University, Fuyang 236037, China; 3 Faculty of Engineering and IT, University of Technology Sydney, Sydney, NSW 2007, Australia; qiaoxi.zhu@gmail.com

**Keywords:** acoustic sensor array, microphone array, linear superarray, sensor array signal processing, differential microphone array, robust beamforming, null constraints, broadband beampattern

## Abstract

Linear superarrays (LSAs) are linear microphone arrays composed of omnidirectional and first-order directional microphones, and their mixed-sensor manifold supports fully steerable differential beamforming. Building on established null-based target-beampattern theory and WNG-constrained beampattern-error design, this paper develops their joint realization for an LSA. Given a look direction and *N* prescribed null offsets, the corresponding *N*th-order differential reference is obtained through the established null-constrained polynomial relation under the stated nonsingularity condition. The LSA weights are then obtained by minimizing the mean-squared beampattern error (MSBE) over the full array plane, subject to exact distortionless and null constraints and a prescribed white noise gain (WNG) lower bound. For the adopted omnidirectional/first-order-directional LSA model, the angular-integral matrices in the MSBE objective are evaluated analytically, while the final weights are obtained from the resulting convex program. Numerical simulations for the evaluated configurations show lower broadband MSBE than the maximum-WNG NC baseline, stable steering, and more consistent broadband patterns than the tested finite-order Jacobi–Anger designs. Anechoic-chamber measurements with an 11-element prototype further confirm that the main lobe, null structure, and steering behavior are retained in measured free-field responses.

## 1. Introduction

Beamforming is a fundamental spatial-filtering technique in radar [1,2], sonar [3,4], and speech acquisition [5,6]. Fixed differential beamformers are particularly attractive for compact acoustic front ends because they can produce high directivity and an approximately frequency-independent spatial response without estimating signal statistics [7,8]. Recent data-driven and neural approaches have also been investigated for microphone-array beamforming, including data-driven WNG selection, neural directional-gain post-filtering, and neural optimization of fixed beamformers under flexible geometric constraints [9,10,11].

Differential beamforming has been studied for linear [8,12], circular [13,14,15], and spherical arrays [16]. A pressure-only linear array, however, senses only the axial projection of the incoming wave, so two directions mirrored with respect to the array axis produce the same phase progression. This front–back ambiguity restricts continuous steering and causes the realized response to change as the look direction moves away from broadside [17,18]. Circular and spherical arrays can supply the missing spatial information, but they usually require a larger physical size; acoustic-vector-sensor arrays provide directional information at each sensor, but at the cost of higher hardware expense and poorer channel consistency [19,20]. Related superarray studies have also considered concentric circular geometries, where omnidirectional and directional sensors are combined to improve steering flexibility and broadband or three-dimensional frequency-invariant beamforming performance [21,22].

A linear superarray (LSA) provides a compact way to overcome these limitations. It combines pressure microphones with first-order directional microphones within a linear aperture, thereby adding directional information without resorting to a large circular or spherical geometry or to a full acoustic-vector sensor at each position. The directional channels break the mirror ambiguity and provide the steering capability needed for fully steerable differential beamforming [23,24]. Existing LSA designs have exploited this structure through Jacobi–Anger (JA) series approximations: a prescribed analytical response is split into omnidirectional and directional components, and the corresponding subarrays are designed to fit these components over all directions. This target-fitting route can provide global pattern control, but it requires the analytical target to be specified a priori, and the approximation error and WNG are affected by finite truncation orders.

Null-constrained (NC) differential beamforming offers a complementary starting point. It specifies the design through the look direction and a finite set of prescribed nulls, which is compact and physically meaningful for interference rejection [25,26,27]. However, this useful specification should be distinguished from the conventional NC solution criterion. The usual minimum-norm, or maximum-WNG, solution enforces the equality constraints only at the selected angles; it does not explicitly preserve the desired frequency-invariant beampattern over the remaining angles, so broadband pattern distortion can still occur.

Motivated by this limitation, this work combines a null-derived reference beampattern with global MSBE minimization, exact look/null constraints, and an explicit WNG lower bound for a mixed-sensor LSA. Table 1 summarizes the closely related studies and clarifies which methodological components of the proposed framework have already been established in the literature. The comparison shows that fully steerable mixed-sensor LSAs were established in [23,24], with anechoic validation also reported in [24]; orientation optimization for WNG equalization was studied in [27]; the null-to-target relation and target-effectiveness issue were developed in [28], and the MSBE–WNG trade-off and broader quadratic or SNR-gain robustness constraints were considered in [29,30,31]. Accordingly, we do not claim any of these individual components as new; rather, our contribution lies in integrating them into an LSA-specific constrained design.

Specifically, the proposed method retains the compact null-based specification of NC designs and uses the established null-to-target relation in [28] to construct the reference beampattern. For a given mixed-sensor LSA, the remaining feasible degrees of freedom are used to minimize the continuous-angle MSBE, while the look-direction and null constraints are enforced exactly, and a prescribed WNG lower bound limits white-noise amplification.

The contributions are as follows. First, we formulate an LSA-specific global angular-domain MSBE design in which the prescribed look direction and nulls remain exact affine constraints and an explicit WNG lower bound limits white-noise amplification. Relative to target-error/WNG methods without exact angular nulls, the mixed-sensor LSA manifold and final exact null constraints are retained; relative to NC robustness methods, the response outside the constrained angles is fitted over the entire angular domain to the null-defined reference. Second, the LSA-specific angular-integral matrices are evaluated analytically, avoiding numerical angular quadrature, and a null-space analysis identifies the degrees of freedom available for global fitting. The final beamformer coefficients are nevertheless obtained from a numerical convex QCQP, rather than from a closed-form weight expression. Third, for an 11-element LSA, we quantify the WNG–MSBE trade-off, steering behavior, and influence of first-order microphone type, and validate the proposed formulation with an anechoic-chamber prototype.

The remainder of this paper is organized as follows. Section 2 defines the LSA model, the NC constraints, and the maximum-WNG NC baseline. Section 3 presents the reference construction, error criterion, and robust formulation. Section 4 and Section 5 report numerical and measured results, respectively.

## 2. Array Model and Null-Constrained Baseline

### 2.1. LSA Signal Model

**Figure 1:**
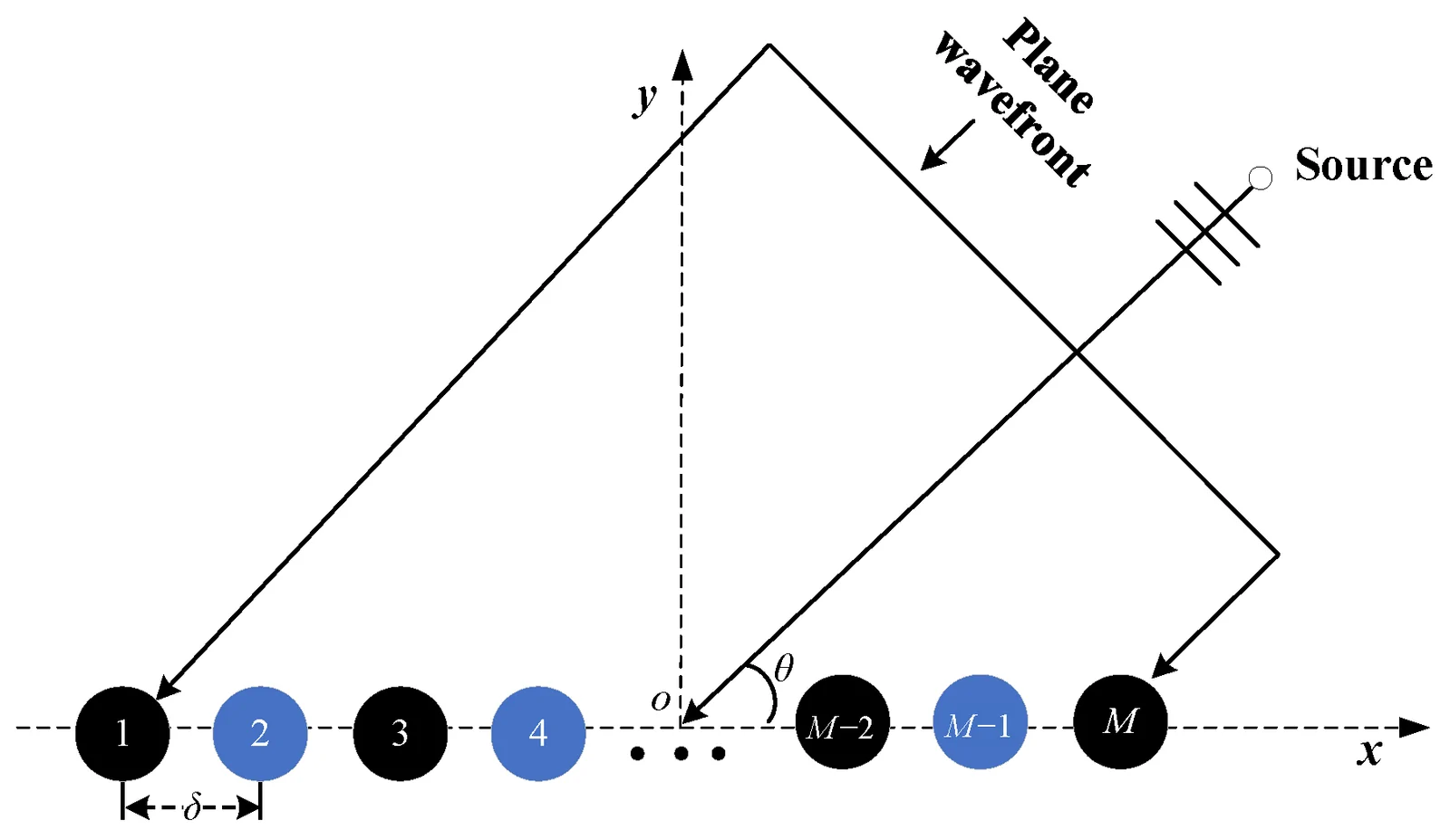
An *M*-element LSA. Black and blue dots denote omnidirectional and first-order directional microphones, respectively. Alternation is used in the prototype but is not required by the formulation.

Consider *M* microphones at positions $x_{m}$ on the *x*-axis, as illustrated in Figure 1. The first-order response of microphone *m* is modeled as

(1) $$g_{m}\left(\theta ;\phi_{m}\right)=a_{m}+\left(1-a_{m}\right)\cos\left(\theta -\phi_{m}\right),$$

where θ is the incident angle in the array plane, $\phi_{m}$ is the microphone orientation, and $a_{m}$ determines the directivity: $a_{m}=1$ is omnidirectional, while $a_{m}=0$, 0.5, 1/3, and $\sqrt{2}-1$ give bidirectional, cardioid, hypercardioid, and supercardioid responses, respectively. The nominal directional microphones in this study point normal to the array, $\phi_{m}=\pi /2$, so that $g_{m}\left(\theta \right)=a_{m}+\left(1-a_{m}\right)\sin\theta$. The alternating layout in Figure 1 follows the prototype; the formulation itself allows arbitrary sensor positions, directivity types, and orientations.

A plane wave impinges on the linear array from direction θ. The resulting steering vector of the *M*-element array is defined as

(2) $$\tilde{d}\left(f,\theta \right)=G\left(\theta \right)d\left(f,\theta \right),$$

where *f* denotes temporal frequency. The orientation arguments are suppressed for brevity. The diagonal matrix G(θ) collects the microphone responses as

(3) $$G\left(\theta \right)=\mathrm{diag}\left[g_{1}\left(\theta \right),g_{2}\left(\theta \right),\ldots ,g_{M}\left(\theta \right)\right].$$

The conventional (omnidirectional) steering vector is

(4) $$d\left(f,\theta \right)={\left[\begin{array}{ccc} e^{j\frac{2\pi f}{c}x_{1}\cos\theta } & \cdots & e^{j\frac{2\pi f}{c}x_{M}\cos\theta } \end{array}\right]}^{T}\;,$$

where *j* is the imaginary unit, c=340 m/s is the speed of sound, and ${\left(\cdot \right)}^{T}$ denotes transpose. For the centered uniform geometry used below, $x_{m}=\left[m-\left(M+1\right)/2\right]\delta$, where δ is the inter-element spacing.

Equations (2)–(4) also show how directional channels remove the mirror ambiguity of a pressure-only linear array. If all microphones are pressure sensors, then $g_{m}=1$, and $\tilde{d}\left(f,\theta \right)=\tilde{d}\left(f,2\pi -\theta \right)$. A directional channel with $\phi_{m}=\pi /2$, however, contains the factor sinθ and therefore gives different responses for this mirror pair.

For a desired signal from the direction $\theta_{s}$, the signals received in the frequency domain are

(5) $$y\left(f\right)=\tilde{d}\left(f,\theta_{s}\right)x\left(f\right)+n(f),$$

where x(f) is the desired signal, and n(f) is the noise vector. The dependence on *f* is omitted hereafter for brevity.

Beamforming aims to extract the desired signal from noisy observations by applying a complex weighting vector h to the received signals y as

(6) $$\begin{array}{cc} z & =h^{H}y\\ \; & =h^{H}\tilde{d}\left(\theta_{s}\right)x+h^{H}n, \end{array}$$

where ${\left(\cdot \right)}^{H}$ denotes conjugate transpose. A distortionless constraint preserves a source from the look direction:

(7) $$h^{H}\tilde{d}\left(\theta_{s}\right)=1.$$

### 2.2. NC Constraints and Maximum-WNG Baseline

This subsection first defines the look-direction and null constraints and then reviews the conventional maximum-WNG solution used as the NC baseline. Following the standard DMA formulation, the frequency-independent directivity pattern of an *N*th-order differential beamformer with its main beam steered toward $\theta_{s}$ can be expressed as [25,26,27]

(8) $$B_{N,\theta_{s}}\left(\theta \right)=\sum_{n=0}^{N}\alpha_{N,n}\cos\left(n\theta -n\theta_{s}\right)=a_{N}^{T}c_{N,\theta_{s}}\left(\theta \right),$$

where

$$\begin{array}{cc} a_{N} & ={\left[\alpha_{N,0},\ldots ,\alpha_{N,N}\right]}^{T}\in R^{N+1},\\ c_{N,\theta_{s}}\left(\theta \right) & ={\left[1,\cos\left(\theta -\theta_{s}\right),\ldots ,\cos N\left(\theta -\theta_{s}\right)\right]}^{T}. \end{array}$$

Unit response in the look direction requires

(9) $$\sum_{n=0}^{N}\alpha_{N,n}=1.$$

Let $0<\beta_{1}<\cdots <\beta_{N}\leq \pi$ denote prescribed null offsets. Because (8) is even about $\theta_{s}$, steering produces nulls at $\theta_{s}\pm \beta_{n}$. The NC specification imposes

(10) $$B\left(h,\theta_{s}\right)=1,\;B\left(h,\theta_{s}\pm \beta_{n}\right)=0,\;n=1,\ldots ,N,$$

where angles are wrapped modulo 2π. Duplicated angles, which occur for example at $\beta_{n}=\pi$, are retained only once. Let $\left\{\vartheta_{1},\ldots ,\vartheta_{L}\right\}$ be the resulting set of distinct constrained angles, ordered with $\vartheta_{1}=\theta_{s}$, and define

(11) $$D_{c}=\left[\begin{array}{c} {\tilde{d}}^{H}\left(\vartheta_{1}\right)\\ \vdots \\ {\tilde{d}}^{H}\left(\vartheta_{L}\right) \end{array}\right]\in C^{L\times M},\;b={\left[1,0,\ldots ,0\right]}^{T}.$$

Since the desired responses are real, (10) is equivalently written as

(12) $$D_{c}h=b.$$

After duplicated or dependent constraints are removed, we assume that the constraint system is feasible and that $D_{c}$ has full row rank. The conventional NC baseline then chooses the minimum-norm feasible solution [26,32]:

(13) $$h_{\mathrm{MN}}=\arg\min_{h}\;{∥h∥}_{2}^{2}\;s.t.\;D_{c}h=b,$$

which has the closed-form expression

(14) $$h_{\mathrm{MN}}=D_{c}^{H}{\left(D_{c}D_{c}^{H}\right)}^{-1}b.$$

### 2.3. Performance Metrics and Limitation of the Maximum-WNG Baseline

We use three standard DMA measures to evaluate the spatial response [5,32]. The beampattern, also called the directivity pattern, describes the response to a unit-amplitude plane wave arriving from angle θ:

(15) $$B\left(h,\theta \right)=h^{H}\tilde{d}\left(\theta \right).$$

The white noise gain (WNG) measures robustness against spatially white sensor noise, while the two-dimensional directivity factor (DF) measures array gain in an isotropic noise field in the array plane. They are defined as

(16) $$\begin{array}{cc} W(h) & =\frac{|h^{H}\tilde{d}\left(\theta_{s}\right){|}^{2}}{h^{H}h}, \end{array}$$

(17) $$\begin{array}{cc} D(h) & =\frac{|B\left(h,\theta_{s}\right){|}^{2}}{{\left(2\pi \right)}^{-1}\int_{0}^{2\pi }{\left|B\left(h,\theta \right)\right|}^{2}d\theta }=\frac{|h^{H}\tilde{d}\left(\theta_{s}\right){|}^{2}}{h^{H}\tilde{\Gamma }h}, \end{array}$$

where

(18) $$\tilde{\Gamma }=\frac{1}{2\pi }\int_{0}^{2\pi }\tilde{d}\left(\theta \right){\tilde{d}}^{H}\left(\theta \right)d\theta .$$

Under the distortionless constraint, $W\left(h\right)=1/{∥h∥}_{2}^{2}$. Therefore, minimizing the coefficient norm in (13) is equivalent to maximizing the WNG, so $h_{\mathrm{MN}}$ is the maximum-WNG NC baseline.

This baseline is robust in the WNG sense, but the equality constraints fix only *L* angular samples: the look direction and the prescribed nulls. The remaining degrees of freedom are used only to reduce ${∥h∥}_{2}$, not to fit the desired frequency-independent directivity pattern at the other angles. Consequently, the DF may increase because the main lobe becomes narrower, even when the overall beampattern deviates from the intended differential response. A high DF alone therefore does not guarantee low pattern error or frequency consistency.

This limitation is complementary to that of series-expansion methods: they fit a prescribed target directivity pattern but are affected by finite truncation orders, whereas the NC specification is simple but its maximum-WNG baseline does not fit the remaining angles [24,26]. This motivates a design that reconstructs the target from the same null specification, fits it globally, and retains an explicit robustness constraint.

## 3. Proposed Robust Global-Error Design

This section turns the NC specification into a robust global-error design. Following the established target-beampattern relation in [28], the prescribed null offsets first define a frequency-independent reference response. The LSA weights are then chosen to approximate this reference over all directions, rather than only at the constrained angles. The original look-direction and null constraints are retained, and a WNG lower bound controls how much of the remaining fitting freedom can be used.

### 3.1. Reference Response Implied by Prescribed Nulls

The prescribed nulls are used here not only as pointwise constraints, but also to define the reference response to be fitted. Substituting the look direction and the prescribed null offsets into (8) gives N+1 linear equations for the N+1 cosine-series coefficients. The look direction gives the normalization row, and the null offsets give *N* zero-response rows:

(19) $$C_{\beta }=\left[\begin{array}{cccc} 1 & 1 & \ldots & 1\\ 1 & \cos\beta_{1} & \ldots & \cos(N\beta_{1})\\ \vdots & \vdots & \; & \vdots \\ 1 & \cos\beta_{N} & \ldots & \cos(N\beta_{N}) \end{array}\right].$$

The first row enforces unit response at the look direction and the remaining rows enforce the *N* null offsets, so

(20) $$C_{\beta }a_{N}=e_{1},\;e_{1}={\left[1,0,\ldots ,0\right]}^{T}.$$

Equation (20) is a cosine-coefficient form of the established null-constrained target relation in [28]; it is not introduced here as a new target-beampattern theory. The cosine–Vandermonde system is nonsingular when $1,\;\cos\beta_{1},\ldots ,\cos\beta_{N}$ are pairwise distinct. Since cosβ is one-to-one on (0,π], distinct offsets satisfy this condition automatically; if absolute null directions are specified, the symmetric pair $\theta_{s}\pm \beta_{n}$ should be counted as one offset. Under this condition, $a_{N}=C_{\beta }^{-1}e_{1}$. A short proof of the nonsingularity condition is given in Appendix A.

### 3.2. Quadratic Global Beampattern Error

To control the response away from the constrained angles, define the mean-squared beampattern error (MSBE) over all angles in the array plane:

(21) $$\epsilon_{N}\left(h\right)=\frac{1}{2\pi }\int_{0}^{2\pi }{\left|B\left(h,\theta \right)-B_{N,\theta_{s}}\left(\theta \right)\right|}^{2}d\theta .$$

Substituting (15) and (8) into (21) gives

(22) $$\epsilon_{N}\left(h\right)=h^{H}\tilde{\Gamma }h-2\;ℜ\;\left\{h^{H}q\right\}+\xi ,$$

where $\tilde{\Gamma }$ is defined in (18), and the vector q and scalar ξ are given by

(23) $$q=\frac{1}{2\pi }\int_{0}^{2\pi }\tilde{d}\left(\theta \right)B_{N,\theta_{s}}^{*}\left(\theta \right)\;d\theta =Qa_{N},$$

(24) $$Q=\frac{1}{2\pi }\int_{0}^{2\pi }\tilde{d}\left(\theta \right)c_{N,\theta_{s}}^{T}\left(\theta \right)\;d\theta ,$$

(25) $$\xi =a_{N}^{T}\bar{C}a_{N},$$

(26) $$\bar{C}=\frac{1}{2\pi }\int_{0}^{2\pi }c_{N,\theta_{s}}\left(\theta \right)c_{N,\theta_{s}}^{T}\left(\theta \right)\;d\theta .$$

Here, q is the projection of the reference response onto the array manifold, and ξ is the reference-response energy. The real-part operator in (22) keeps the quadratic objective real. The closed-form entries of $\tilde{\Gamma }$, Q, and $\bar{C}$ are derived in Appendix B. Their analytical evaluation avoids numerical angular quadrature. These closed-form matrices should not be confused with a closed-form beamformer: the final coefficient vector is obtained by solving (30).

### 3.3. WNG-Constrained Global-Error Formulation

The proposed design replaces the minimum-norm criterion in (13) by the MSBE objective in (22), while keeping the same look-direction and null constraints. To retain robustness, the design also imposes a WNG lower bound. Under the distortionless constraint, let $\zeta_{\mathrm{WNG},\mathrm{dB}}$ denote the prescribed WNG lower bound expressed in decibels. The WNG constraint is then equivalent to

(27) $$h^{H}h\leq 10^{-\zeta_{\mathrm{WNG},\mathrm{dB}}/10},$$

The lower bound is specified relative to the maximum-WNG NC baseline. Since the largest feasible WNG is attained by $h_{\mathrm{MN}}$, define

(28) $$W_{\max,\mathrm{dB}}=10\log_{10}\;\left(\frac{1}{h_{\mathrm{MN}}^{H}h_{\mathrm{MN}}}\right),$$

where $h_{\mathrm{MN}}$ is given in (14), and set

(29) $$\zeta_{\mathrm{WNG},\mathrm{dB}}=W_{\max,\mathrm{dB}}-v,\;v\geq 0,$$

Combining (22), (12), and (27) gives the improved null-constrained (INC) design

(30) $$\begin{array}{cc} \min_{h}\;\; & h^{H}\tilde{\Gamma }h-2\;ℜ\;\left\{h^{H}q\right\}+\xi \\ s.t.\;\; & D_{c}h=b,\\ \; & h^{H}h\leq 10^{-\zeta_{\mathrm{WNG},\mathrm{dB}}/10}. \end{array}$$

The parameter *v*, expressed in decibels, is a user-selected WNG-loss allowance relative to the maximum-WNG NC solution, not an additional robustness metric or a methodological novelty. The primary robustness specification remains the absolute lower bound $\zeta_{\mathrm{WNG},\mathrm{dB}}$ in (27); (29) merely provides a convenient way to set that bound for a given array. When v=0, the norm ball touches the feasible equality set only at $h_{\mathrm{MN}}$, so INC reduces to NC. Increasing *v* allows a lower MSBE at the cost of a smaller guaranteed WNG.

This trade-off can be interpreted through the null space of the NC constraints. Let $Z\in C^{M\times (M-r)}$ contain an orthonormal basis for $N(D_{c})$, where $r=\mathrm{rank}(D_{c})$. Any beamformer satisfying the same equality constraints can be written as

(31) $$h=h_{\mathrm{MN}}+Zu,\;u\in C^{M-r}.$$

The NC baseline corresponds to u=0, whereas INC uses nonzero null-space components to reduce the response error at unconstrained angles. Thus, only the M−r null-space dimensions are available for global pattern fitting; when M=r, the equality constraints leave no freedom beyond the NC baseline.

Because $\tilde{\Gamma }$ is positive semidefinite by construction, the objective is convex; the equality set and norm ball are also convex. Thus, (30) is a standard convex quadratically constrained quadratic program and can be solved with established software such as CVX 2.2 [33]. The contribution is not any one of the established ingredients in isolation. It is their LSA-specific integration: the null-defined reference is fitted continuously over the array plane, the same look/null specification remains exact in the final weights, and the remaining feasible degrees of freedom are controlled by a WNG lower bound.

The robustness role of the WNG bound follows from the standard perturbation interpretation of WNG in robust and superdirective beamforming [34,35]. Recent robust HD and differential beamforming studies have further considered data-driven WNG constraints, beampattern-error criteria, structured noise models, and generalized SNR-gain constraints [9,29,30,31]. Suppose the actual steering vector is $\tilde{d}\left(\theta \right)+\Delta \tilde{d}\left(\theta \right)$. The induced response error satisfies

(32) $$\left|\Delta B\left(\theta \right)|=\right|h^{H}\Delta \tilde{d}{\left(\theta \right)|\leq ∥h∥}_{2}∥\Delta \tilde{d}{\left(\theta \right)∥}_{2}\leq 10^{-\zeta_{\mathrm{WNG},\mathrm{dB}}/20}{∥\Delta \tilde{d}\left(\theta \right)∥}_{2}.$$

Thus, a larger WNG lower bound limits the worst-case amplification of steering-vector mismatch. In (30), this established WNG control is coupled directly with global beampattern-error minimization; *v* only re-expresses the selected absolute WNG threshold relative to the maximum-WNG NC baseline.

## 4. Simulations

**Figure 2:**
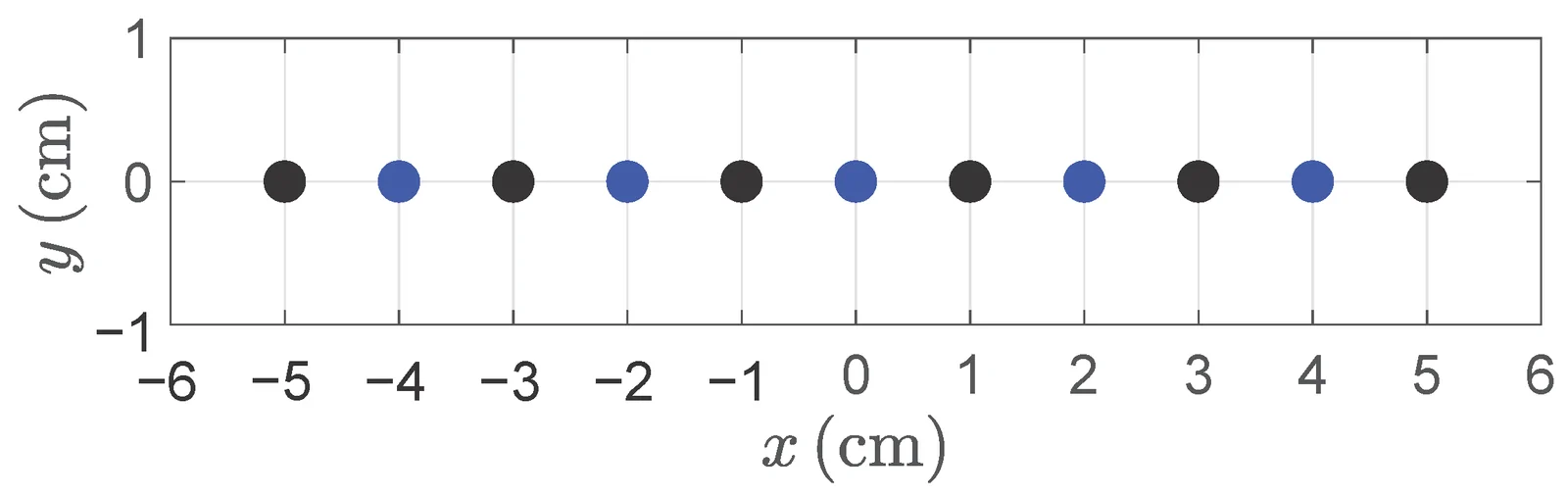
Geometry of the uniform LSA used in simulations: black dots denote omnidirectional microphones, and blue dots denote bidirectional microphones.

Numerical simulations are used to verify the mechanism of the proposed design and to compare it with the NC baseline and the JA expansion-based method. Unless stated otherwise, the 11-element LSA contains six omnidirectional and five bidirectional microphones in an alternating uniform layout with δ=0.01 m (Figure 2); all bidirectional axes are normal to the array. The sensor count is kept fixed so that the observed differences come from the design criteria rather than from aperture or channel-count changes. Performance is reported using DF, WNG in decibels, and MSBE in decibels, $10\log_{10}\epsilon_{N}$. Unless otherwise stated, all simulated broadband plots are evaluated from 0.2 to 5 kHz using 481 uniformly spaced frequency points with a spacing of 10 Hz.

### 4.1. Broadband Comparison with the NC Baseline

**Figure 3:**
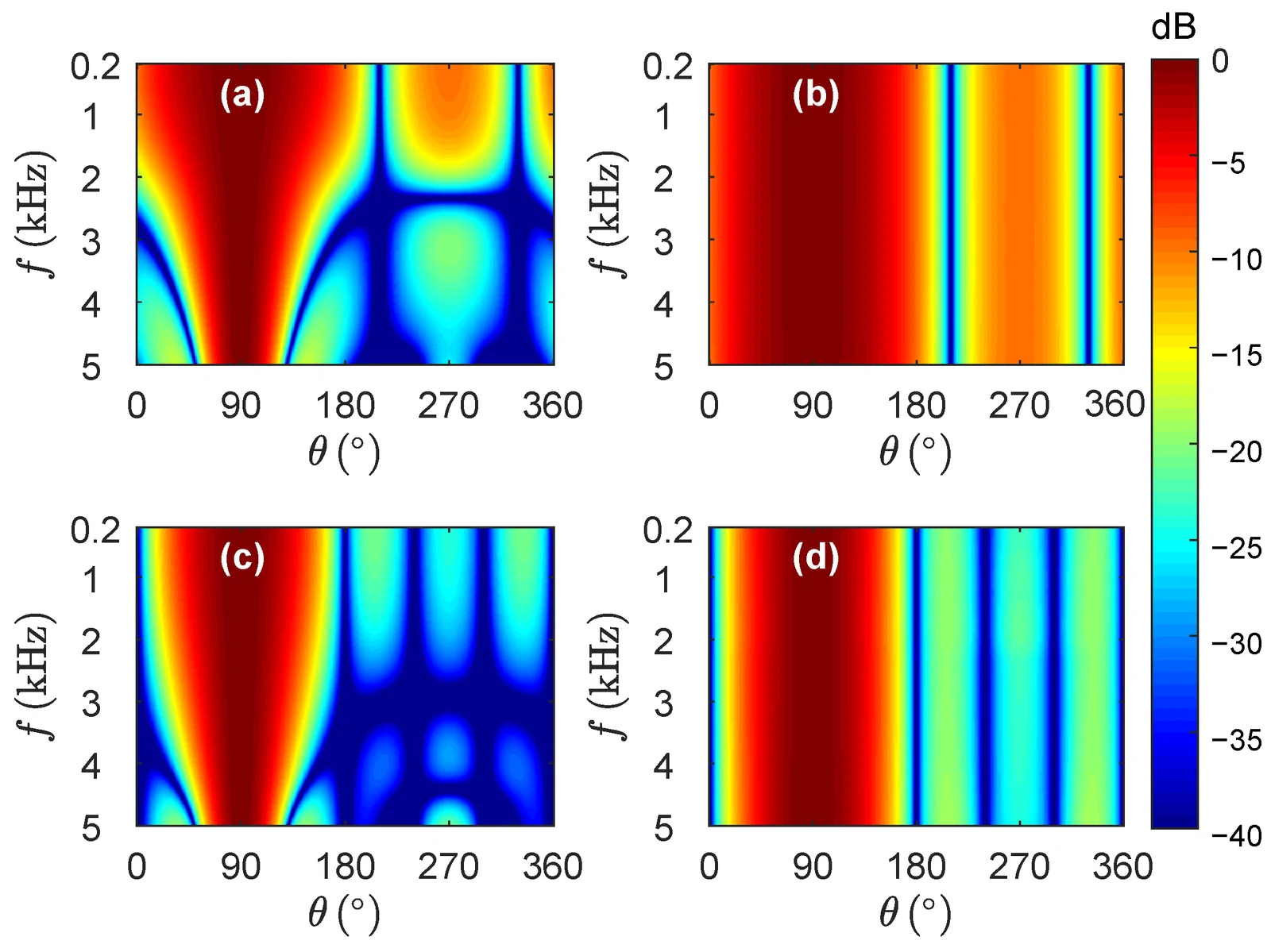
Broadband beampatterns for $\theta_{s}=90{}^{\circ}$ designed by NC and INC: (**a**) N=1 NC, (**b**) N=1 INC, (**c**) N=2 NC, (**d**) N=2 INC.

Figure 3 compares NC and INC for N=1 and N=2 at $\theta_{s}=90{}^{\circ}$. NC satisfies the look-direction and null constraints exactly, but its main lobe narrows as the frequency increases, and its unconstrained sidelobes change. INC uses the same equality constraints while allowing a 10 dB WNG loss relative to the maximum-WNG NC baseline (v=10 dB), and it more closely preserves the reference response across frequency.

**Figure 4:**
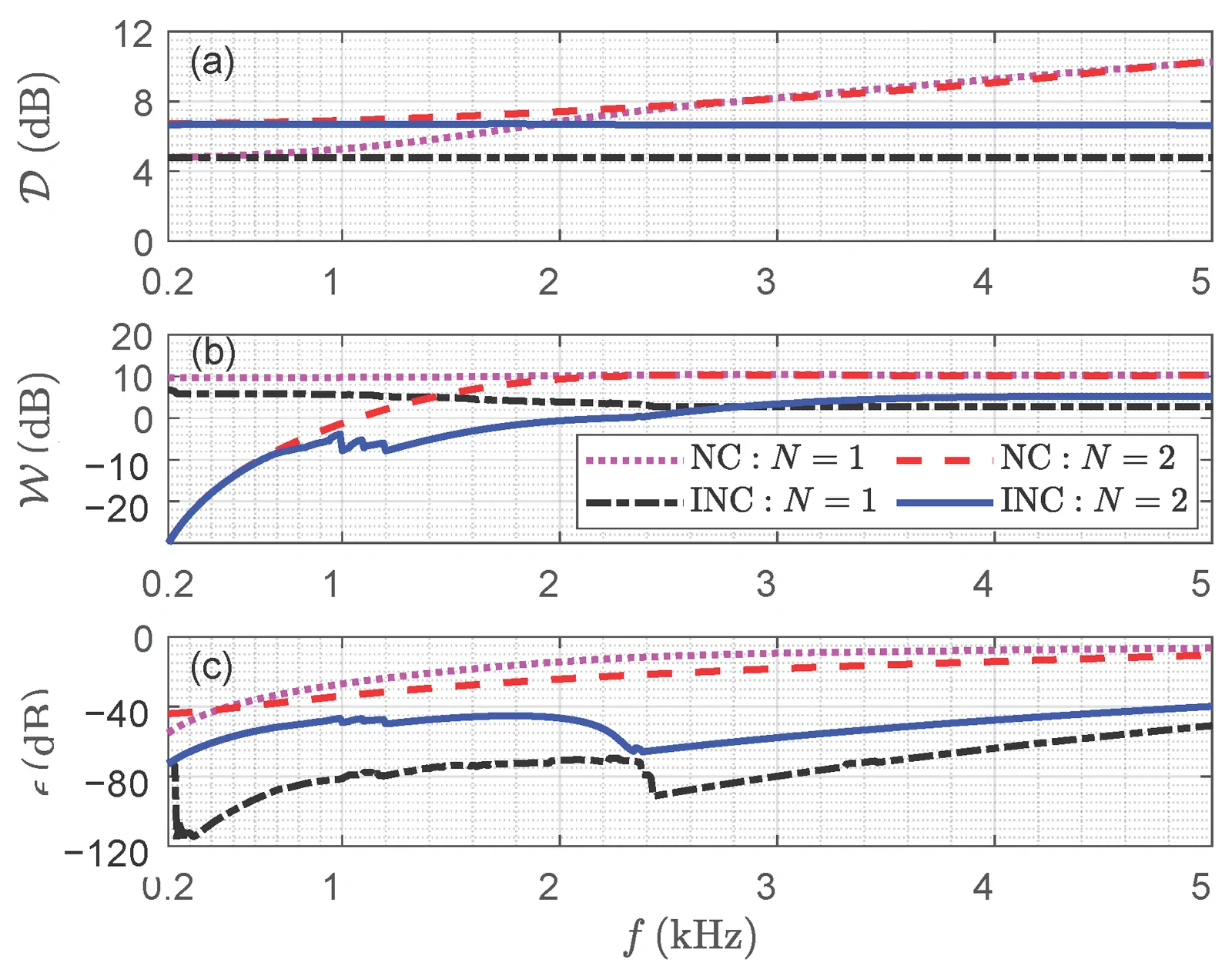
Performance of NC and INC for N=1 and N=2: (**a**) D, (**b**) W, and (**c**) MSBE ϵ.

Figure 4 quantifies the same behavior. The NC solution obtains a larger DF at high frequency, but its MSBE is also much larger. The larger DF is therefore mainly a consequence of response narrowing, not better agreement with the desired differential pattern. INC lowers the MSBE by using part of the WNG margin for global fitting.

Thus, exact nulls and a high DF are insufficient to establish a frequency-invariant differential response; the global error metric is necessary to diagnose and correct the response between constrained angles.

### 4.2. Controlled Robustness–Accuracy Trade-Off

**Figure 5:**
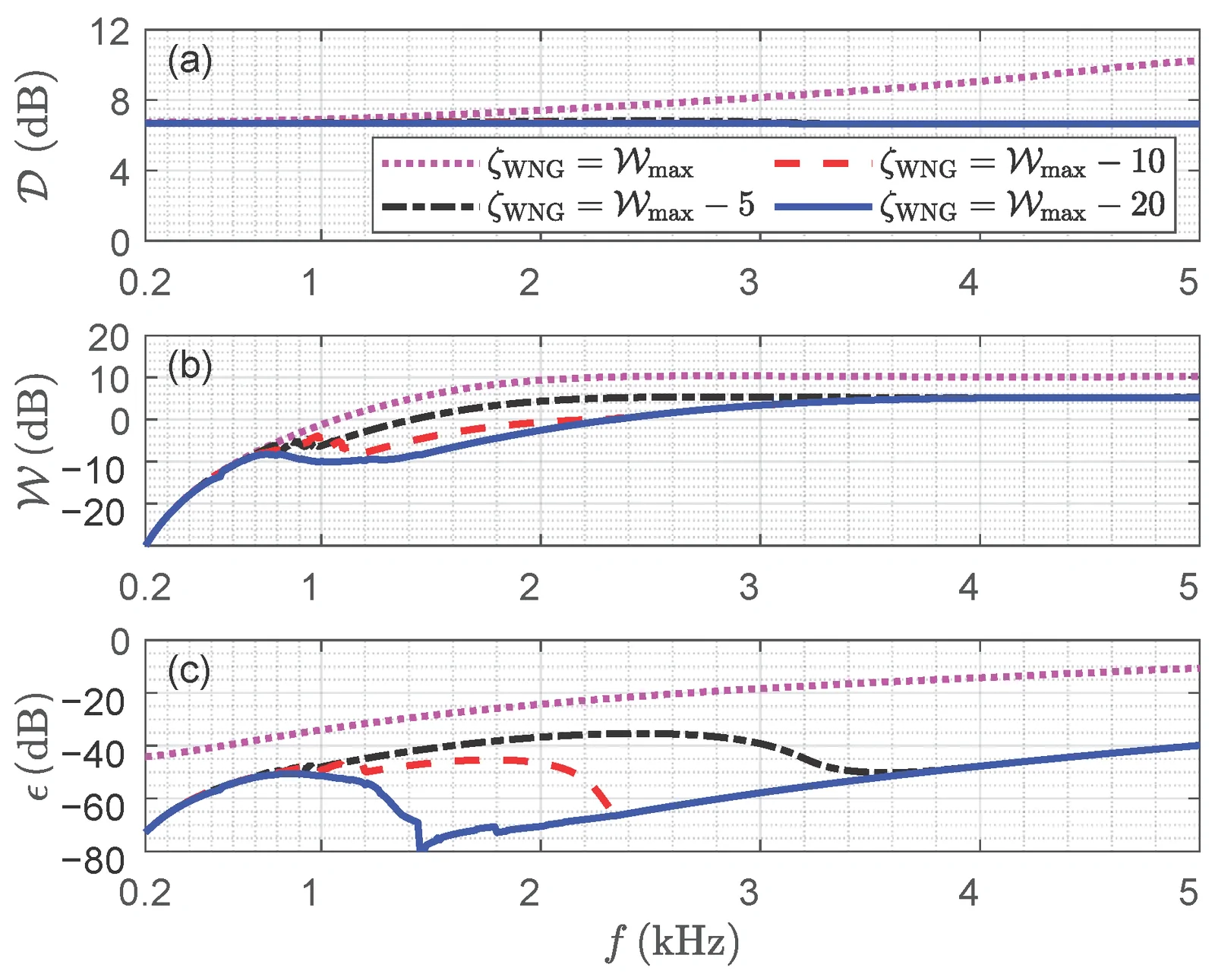
INC performance for N=2 under different WNG thresholds: (**a**) D, (**b**) W, and (**c**) MSBE ϵ.

We next examine the effect of the WNG threshold for the N=2 INC design. Figure 5 compares v∈{0, 5, 10, 20} dB, or equivalently, $\zeta_{\mathrm{WNG},\mathrm{dB}}=W_{\max,\mathrm{dB}}-v$. The case v=0 is the NC limit, because the WNG bound is tight at the minimum-norm feasible solution. As *v* increases, the feasible norm ball expands, the MSBE decreases in Figure 5c, and the guaranteed WNG decreases in Figure 5b. For the subsequent INC-only steering, microphone-type, and experimental evaluations, we use v=10 dB because it keeps the simulated MSBE below −40 dB over the evaluated band without the larger WNG loss observed at v=20 dB. This is an operating choice for the present array, not a universal threshold. The JA–INC comparison in Section 4.5 instead uses the WNG-matched bound specified there.

To examine whether this WNG–accuracy trade-off is retained under microphone mismatch, we performed a fixed-weight Monte Carlo sensitivity test at 1, 3, and 5 kHz for v=5, 10, and 20 dB. For each nominal design, the weights were calculated once and held fixed over 5000 realizations. Each realization jointly applied independent uniformly distributed gain (±5%), phase (±5°), and axial-position (±0.5 mm) errors to all microphones, together with orientation (±5°) and directivity-parameter (±0.05) errors to the bidirectional microphones. These ranges are representative sensitivity-test levels rather than universal manufacturing tolerances. The perturbed complex beampattern was evaluated directly using the MSBE in (21), without look-direction renormalization.

Table 2 confirms the expected trade-off. At 1 kHz, increasing *v* lowers the nominal MSBE but increases the mean MSBE under joint mismatch. At 3 and 5 kHz, v=10 and 20 dB produce essentially the same results because the WNG constraint is already inactive at v=10 dB. Thus, for the evaluated array, v=10 dB improves the nominal accuracy relative to v=5 dB, whereas increasing it to 20 dB provides no additional benefit at 3 and 5 kHz and increases low-frequency mismatch sensitivity. This supports v=10 dB as a practical robustness–accuracy compromise for the present configuration, rather than as a universal value. In general, when an application specifies a minimum acceptable WNG, $\zeta_{\mathrm{WNG},\mathrm{dB}}$ should be set directly to that requirement and $v=W_{\max,\mathrm{dB}}-\zeta_{\mathrm{WNG},\mathrm{dB}}$. When no absolute WNG requirement is available, a small set of candidate values can be evaluated, and the smallest *v* satisfying both the nominal-MSBE requirement and a representative mismatch-tolerance requirement can be selected.

### 4.3. Full Steerability

**Figure 6:**
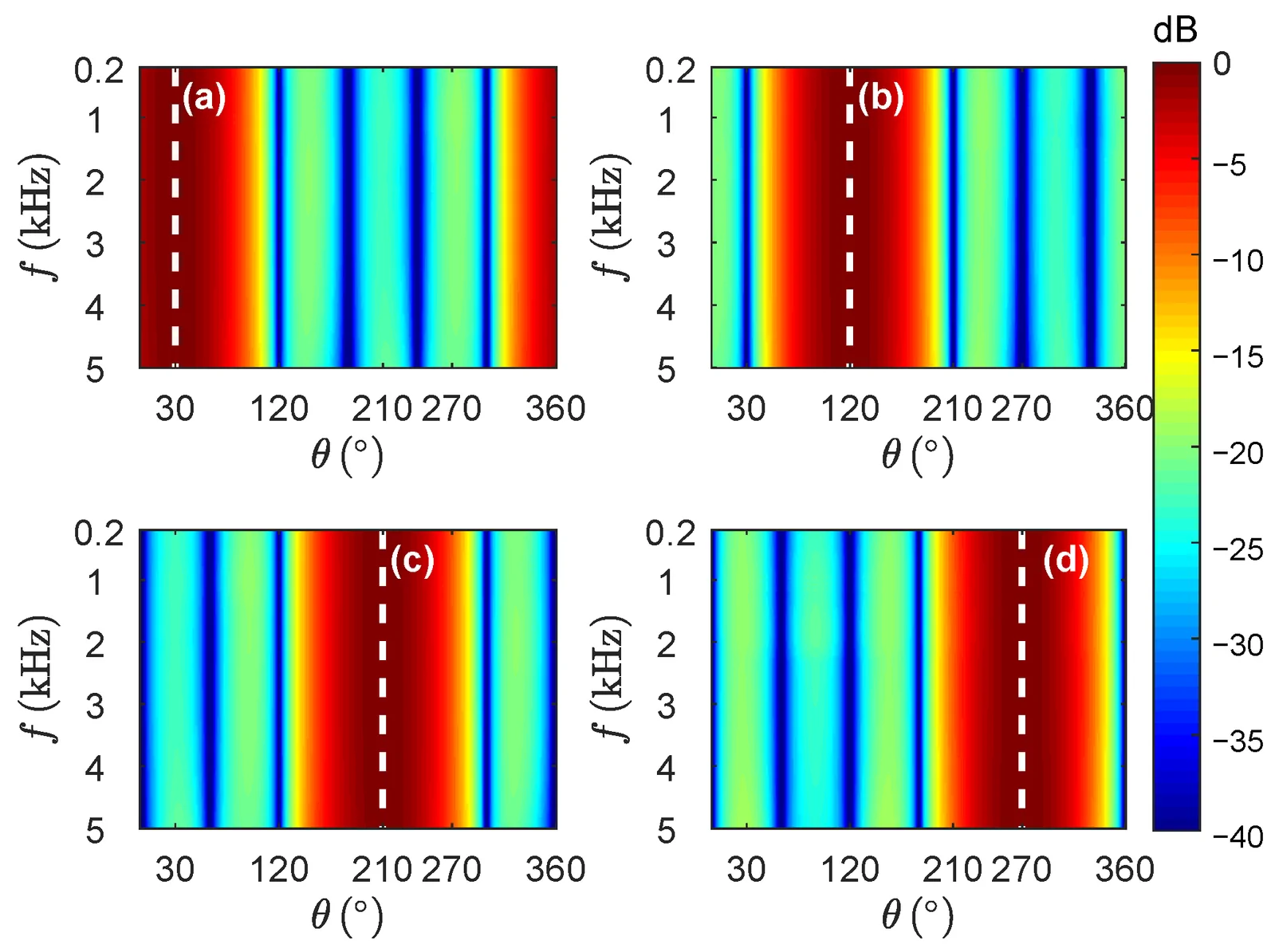
Broadband N=2 beampatterns designed by INC for (**a**) $\theta_{s}=30{}^{\circ}$, (**b**) $\theta_{s}=120{}^{\circ}$, (**c**) $\theta_{s}=210{}^{\circ}$, and (**d**) $\theta_{s}=270{}^{\circ}$.

We evaluate steerability for N=2 by steering the main lobe to four representative directions: $\theta_{s}=30{}^{\circ}$, 120°, 210°, and 270°, while enforcing symmetric null-pair constraints at $\theta_{s}\pm 90{}^{\circ}$ and $\theta_{s}\pm 150{}^{\circ}$. Figure 6 shows that the main lobe tracks $\theta_{s}$ and the broadband pattern remains close to the prescribed response under steering.

**Figure 7:**
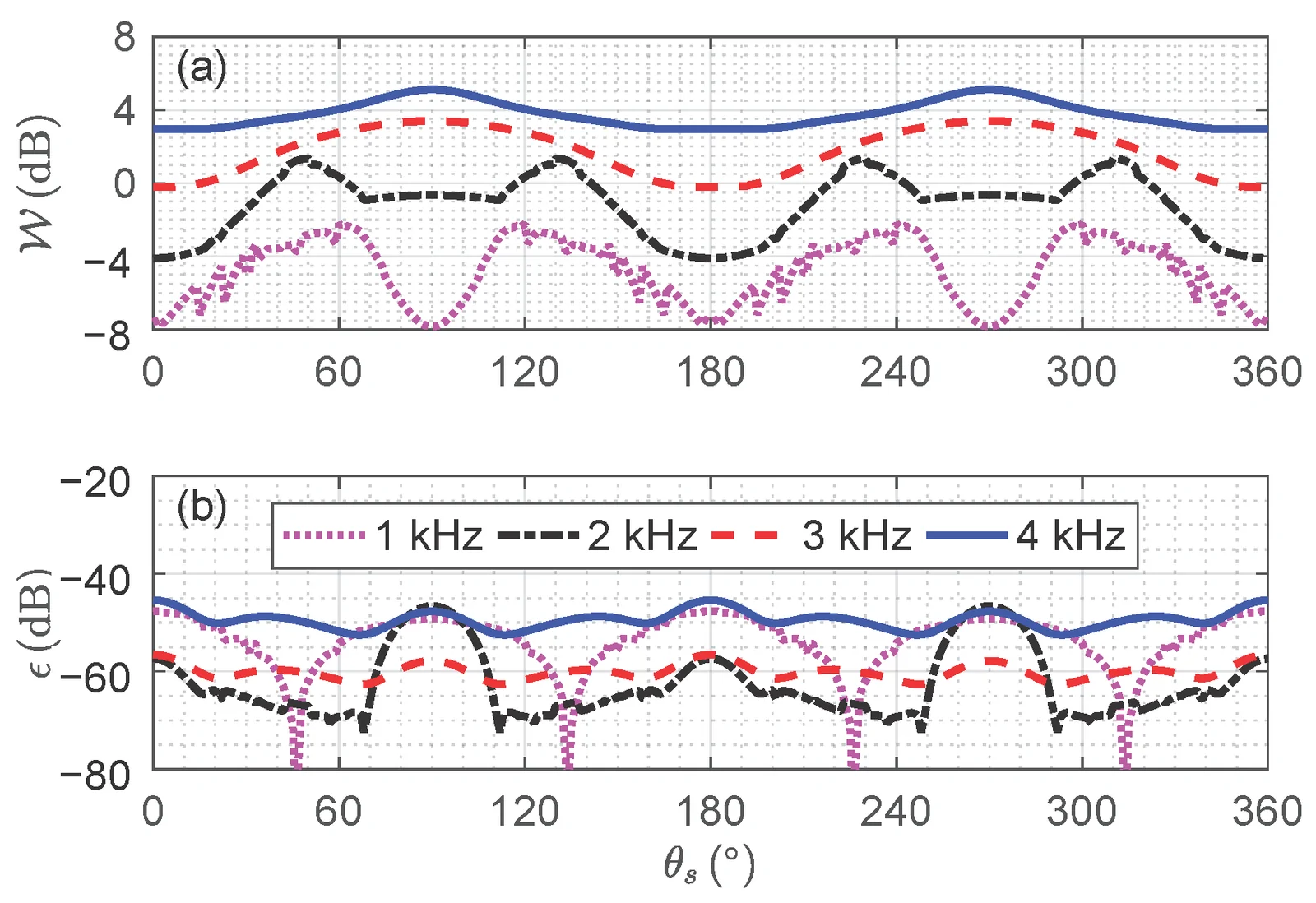
Steering -angle dependence for N=2 at 1, 2, 3, and 4 kHz: (**a**) WNG and (**b**) MSBE. Full steerability denotes angular coverage, not constant WNG.

To distinguish steering coverage from exact performance invariance, Figure 7 plots both WNG and MSBE versus $\theta_{s}$ at 1, 2, 3, and 4 kHz. The look direction covers all angles in the array plane and the MSBE remains below −40 dB, but WNG still varies with angle and frequency. This is why the method is described as fully steerable rather than “steering invariant.”

### 4.4. Influence of First-Order Microphone Type

**Figure 8:**
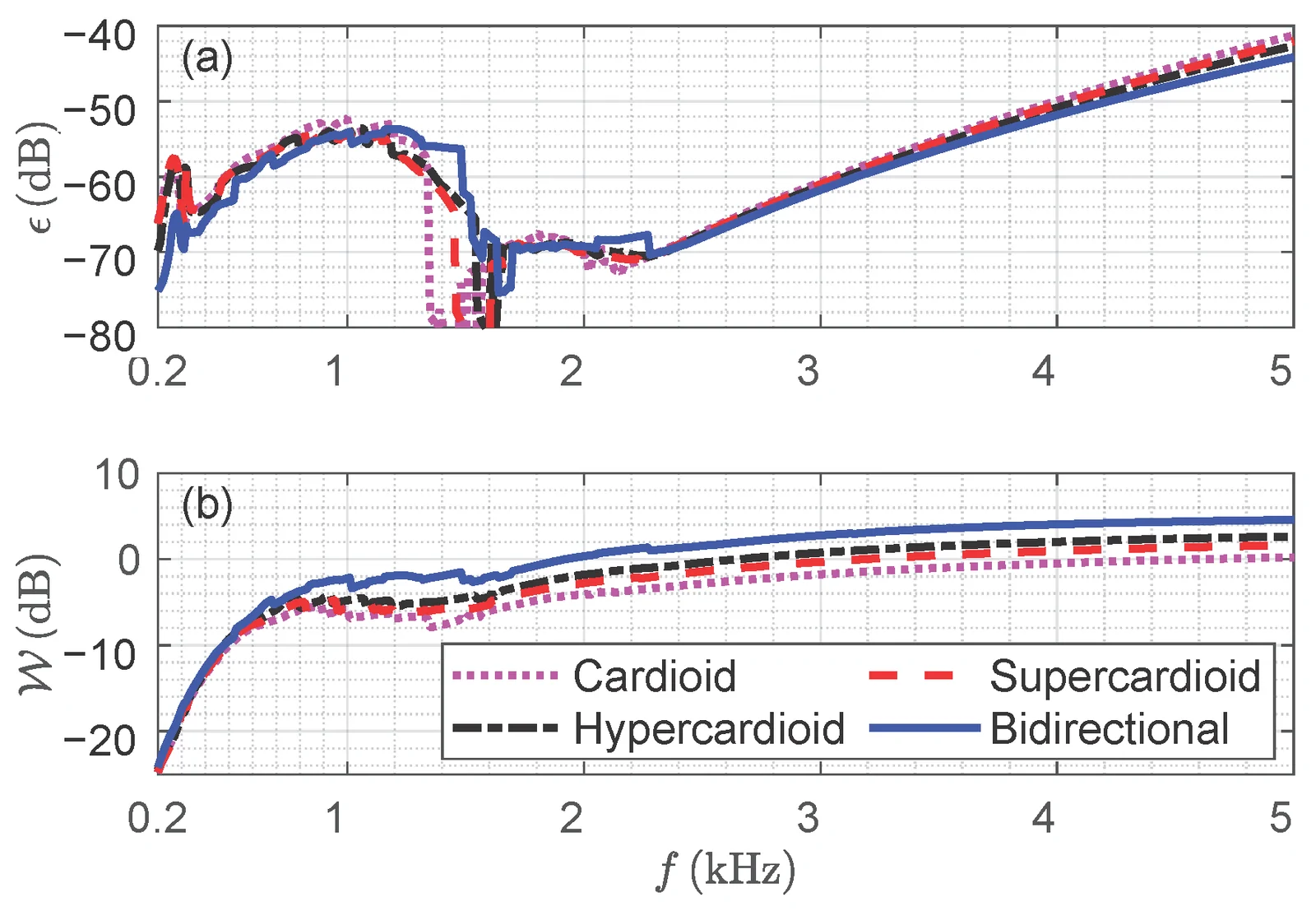
Effect of the directional microphone type for the N=2 INC design: (**a**) MSBE and (**b**) WNG.

The proposed optimization is not tied to bidirectional elements. Figure 8 replaces the five directional microphones by cardioid, supercardioid, hypercardioid, or bidirectional microphones while keeping the same array geometry, second-order design, $\theta_{s}=90{}^{\circ}$, and v=10 dB. All four types attain similar MSBE over most of the band, whereas the bidirectional case provides the highest WNG above approximately 800 Hz. Hence, bidirectional microphones were selected for the prototype because of their robustness in this configuration, not because the formulation requires this sensor type.

### 4.5. Comparison with the Jacobi–Anger Expansion-Based Method

We compare INC with the JA expansion-based LSA method [24]. Both methods use the 11-element array in Figure 2 with δ=0.01 m and the frequency grid f=0.2:0.01:5 kHz. Following [24], the common target is a second-order cardioid with $\left(\alpha_{2,0},\alpha_{2,1},\alpha_{2,2}\right)=\left(1/4,\;1/2,\;1/4\right)$ steered to $\theta_{s}=60{}^{\circ}$. For INC, the offsets {90°,180°} reconstruct exactly these coefficients through (19) and yield the three distinct null directions 150°, 240°, and 330°. JA is evaluated with $\left(N_{1},N_{2}\right)=\left(2,\;1\right),\;\left(3,\;2\right)$, and (4, 3). For the second-order target, (2, 1) is the minimum admissible pair in the JA formulation, while (3, 2) and (4, 3) are two successively higher pairs used in the truncation-order study of [24]. No global optimality is claimed; (4, 3) is only the best-performing configuration among the three tested pairs for the present array and frequency range. It is therefore used as the JA reference for robustness matching. The INC WNG bound is adjusted independently at each frequency so that INC and JA (4, 3) attain similar WNG values.

**Figure 9:**
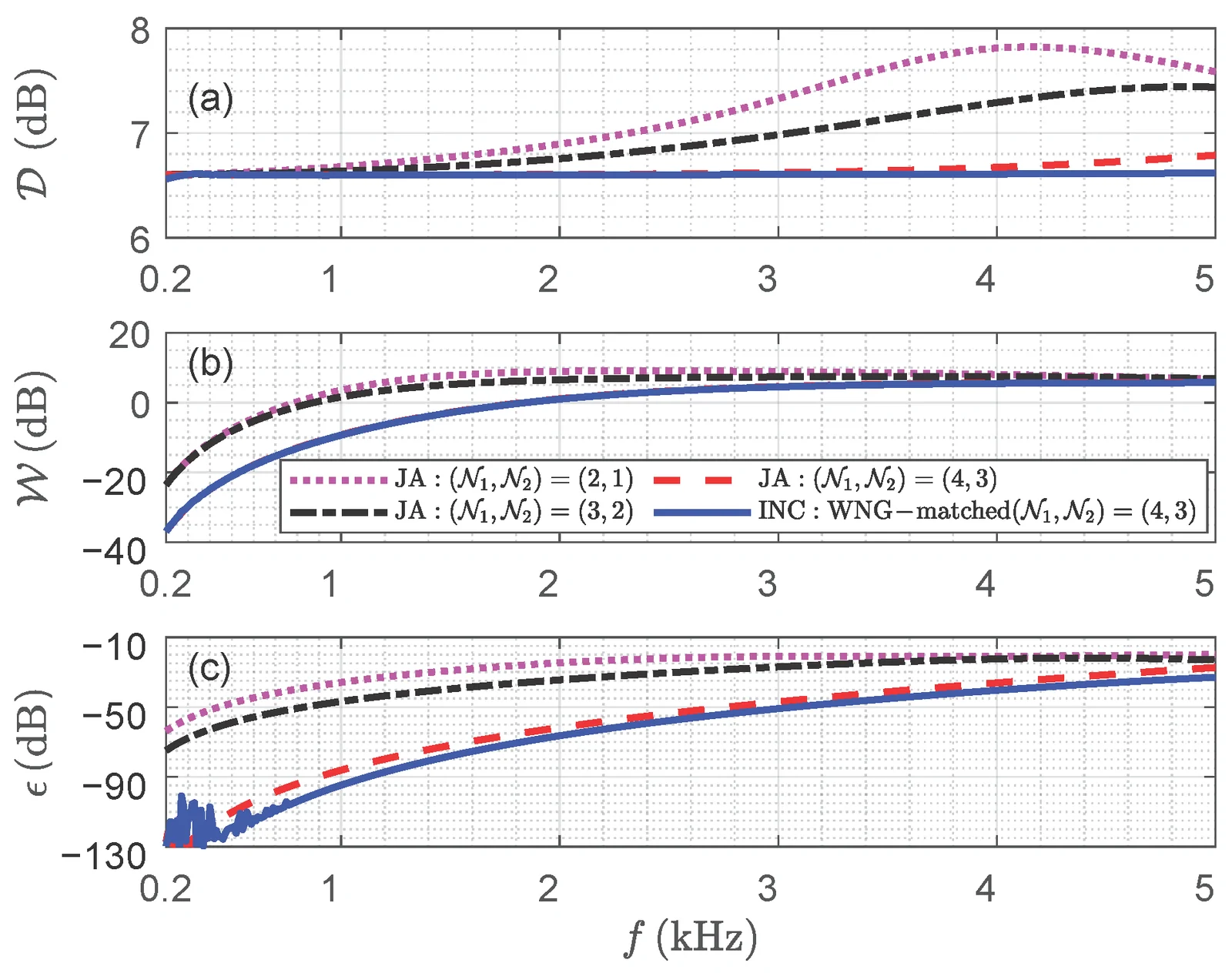
Comparison between JA [24] with $\left(N_{1},N_{2}\right)=\left(2,\;1\right),\;\left(3,\;2\right),\;\left(4,\;3\right)$ and the WNG-matched INC result for a second-order cardioid target at $\theta_{s}=60{}^{\circ}$: (**a**) D, (**b**) W, and (**c**) MSBE ϵ. In panel (**b**), the red dashed JA (4, 3) curve is largely obscured by the blue INC curve because of their close agreement.

**Figure 10:**
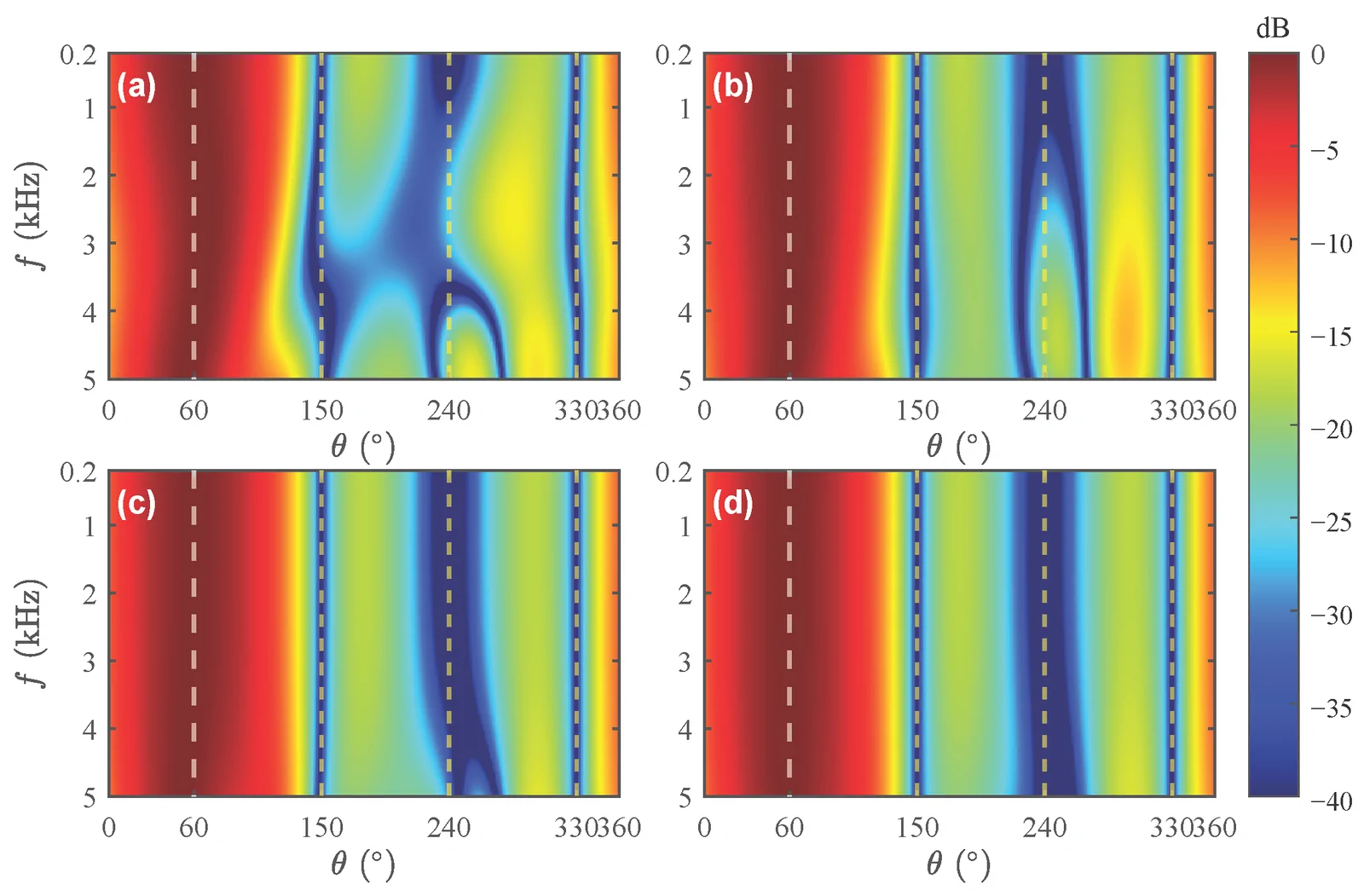
Broadband beampatterns for second-order cardioid approximation: (**a**–**c**) the JA method [24] with $\left(N_{1},N_{2}\right)=\left(2,\;1\right)$, (3, 2), and (4, 3); (**d**) INC WNG-matched to JA (4, 3). The white dashed line marks the look direction $\theta_{s}=60{}^{\circ}$. The yellow dashed lines indicate the prescribed null directions at 150°, 240°, and 330°.

Figure 9 and Figure 10 display the full simulated frequency range of 0.2–5 kHz. Figure 9a shows that the DF curves of JA (4, 3) and INC nearly overlap and remain almost frequency independent, whereas the lower-order JA configurations exhibit larger frequency-dependent variations. Figure 9b confirms that JA (4, 3) and INC achieve similar WNG values. Figure 9c shows that increasing the JA truncation orders reduces its MSBE; nevertheless, over 0.5–5 kHz and under the matched WNG, INC provides a lower MSBE than the best tested JA configuration, (4, 3).

Figure 10 further illustrates the corresponding broadband spatial responses. As shown in Figure 10a–c, increasing the JA truncation orders improves the frequency consistency of the approximated beampattern, with JA (4, 3) giving the best result among the three tested configurations. Compared with JA (4, 3) at a similar WNG, INC, shown in Figure 10d, maintains the prescribed look and null directions and provides a more frequency-consistent approximation to the desired second-order cardioid, in agreement with the MSBE comparison in Figure 9c.

In addition to comparing their performance, we assessed the computational cost of offline weight design. In terms of computational complexity, JA uses direct minimum-norm matrix solutions, whereas INC solves a convex program iteratively using CVX 2.2 (CVX Research, Inc., Austin, TX, USA) with its bundled SeDuMi 1.3.4 solver (Jos F. Sturm, Tilburg University, Tilburg, The Netherlands). The effect of these different solution procedures is reflected in the measured design times. On an Intel Core i7-10700 CPU (Intel Corporation, Santa Clara, CA, USA) running MATLAB R2023a (The MathWorks, Inc., Natick, MA, USA), the WNG-matched JA (4, 3) and INC designs required approximately 0.00065 s and 0.35 s at 1 kHz, respectively. For the complete broadband design for one prescribed steering direction, $\theta_{s}=60{}^{\circ}$, the corresponding total times over the 481 frequency points from 0.2 to 5 kHz were approximately 0.42 s and 168 s. Thus, the reported 168 s corresponds to generating the complete set of frequency-dependent INC weights for this single look direction and does not include a sweep over multiple steering directions; the angular sweep elsewhere in the manuscript is used only to evaluate steerability. For the same 481-frequency comparison, all JA solutions yielded finite coefficient vectors, while CVX 2.2/SeDuMi returned the status *Solved* for all 481 INC optimization problems, with no *Inaccurate* status, numerical failure, or infeasibility warning. These results indicate stable observed numerical behavior for the evaluated configuration. Thus, JA is more computationally efficient in calculating the beamformer coefficients. For applications in which the beamformer coefficients can be computed and stored in advance, however, the additional offline cost of INC does not affect real-time filtering because only the precomputed weights are used during operation.

## 5. Experiment and Discussion

The simulations above use analytical free-field steering vectors. The experiment evaluates whether the resulting weights retain the intended spatial response when applied to measured transfer functions of a physical prototype. Thus, this section provides an anechoic, offline beampattern validation of the 11-element LSA prototype.

### 5.1. Experimental Setup

**Figure 11:**
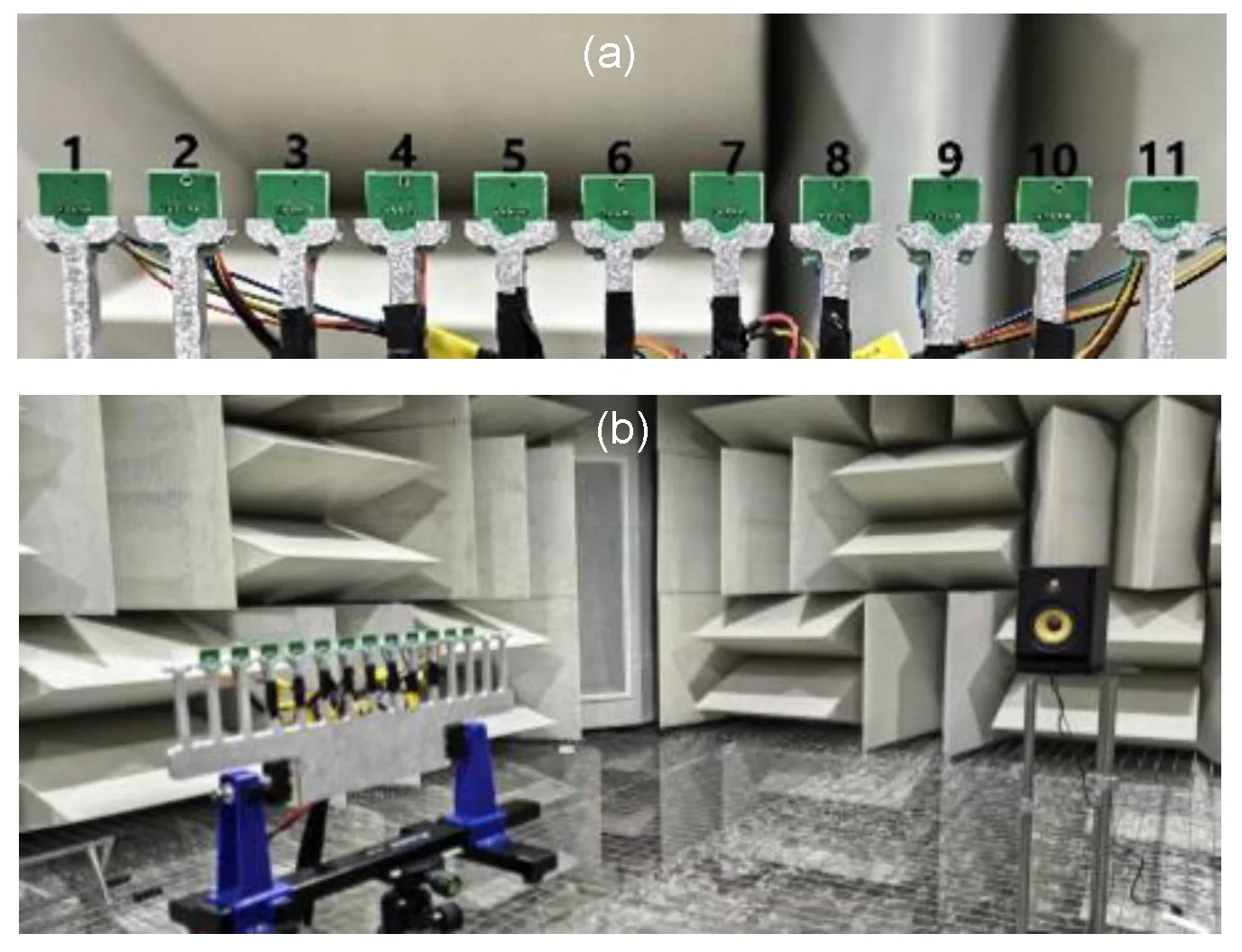
Experimental setup: (**a**) an 11-element interleaved LSA consisting of omnidirectional and bidirectional microphones; (**b**) the array mounted on a turntable with a loudspeaker positioned 3 m away in an anechoic chamber.

Experiments were conducted in a 9.0 m × 9.6 m × 5.1 m anechoic chamber. The prototype comprises six omnidirectional microphones (SPW0878LR5H-1, Knowles Electronics, LLC, Itasca, IL, USA) and five bidirectional microphones (SKR0600, Soundskrit, Montreal, QC, Canada) with δ=0.02 m. This spacing differs from the numerical setting because of prototype hardware constraints, and the experimental weights are calculated using the prototype spacing. Figure 11a shows the interleaved LSA. As shown in Figure 11b, the array was mounted on a turntable, and a loudspeaker (Rokit7, KRK Systems, Nashville, TN, USA) was placed 3 m from the array center at a height of 1.6 m. A logarithmic sweep from 20 Hz to 20 kHz was generated and reproduced via an audio interface (Fireface UFX III, RME/IMM electronics GmbH, Mittweida, Germany); the received signals were recorded through the same interface to estimate the loudspeaker-to-microphone transfer functions. The array was rotated from 0° to 360° in 5° steps, giving 73 measured angles, including both endpoints. The measured transfer functions were then combined with the designed weights to compute offline beampatterns. For the measured broadband maps, the estimated transfer functions and the corresponding beamformer weights were evaluated on the same 0.2–5 kHz grid with 10 Hz spacing, giving 481 frequency points.

### 5.2. Measured Beampattern Results

**Figure 12:**
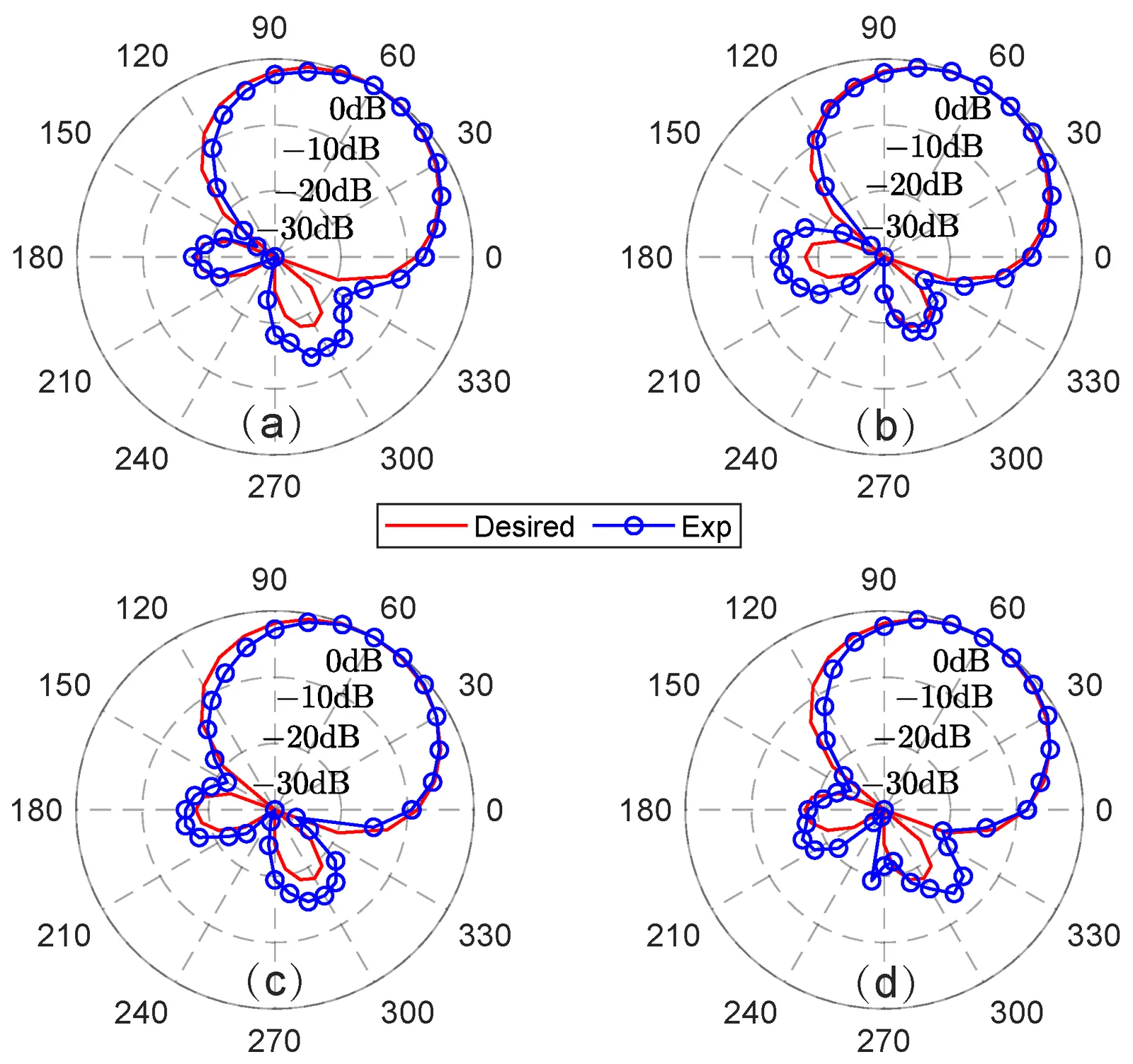
Measured (offline) and desired beampatterns for second-order cardioid approximation with $\theta_{s}=60{}^{\circ}$: (**a**) 500 Hz, (**b**) 1 kHz, (**c**) 2 kHz, and (**d**) 3 kHz.

We first evaluate the same second-order cardioid target used in Section 4.5: $\theta_{s}=60{}^{\circ}$, null offsets {90°,180°}, and v=10 dB. Figure 12 compares the reference and measured offline beampatterns. The measured patterns retain the principal lobe and constrained null structure from 500 Hz to 3 kHz. Although small deviations from the reference are visible, they do not obscure the main spatial features of the designed response.

**Figure 13:**
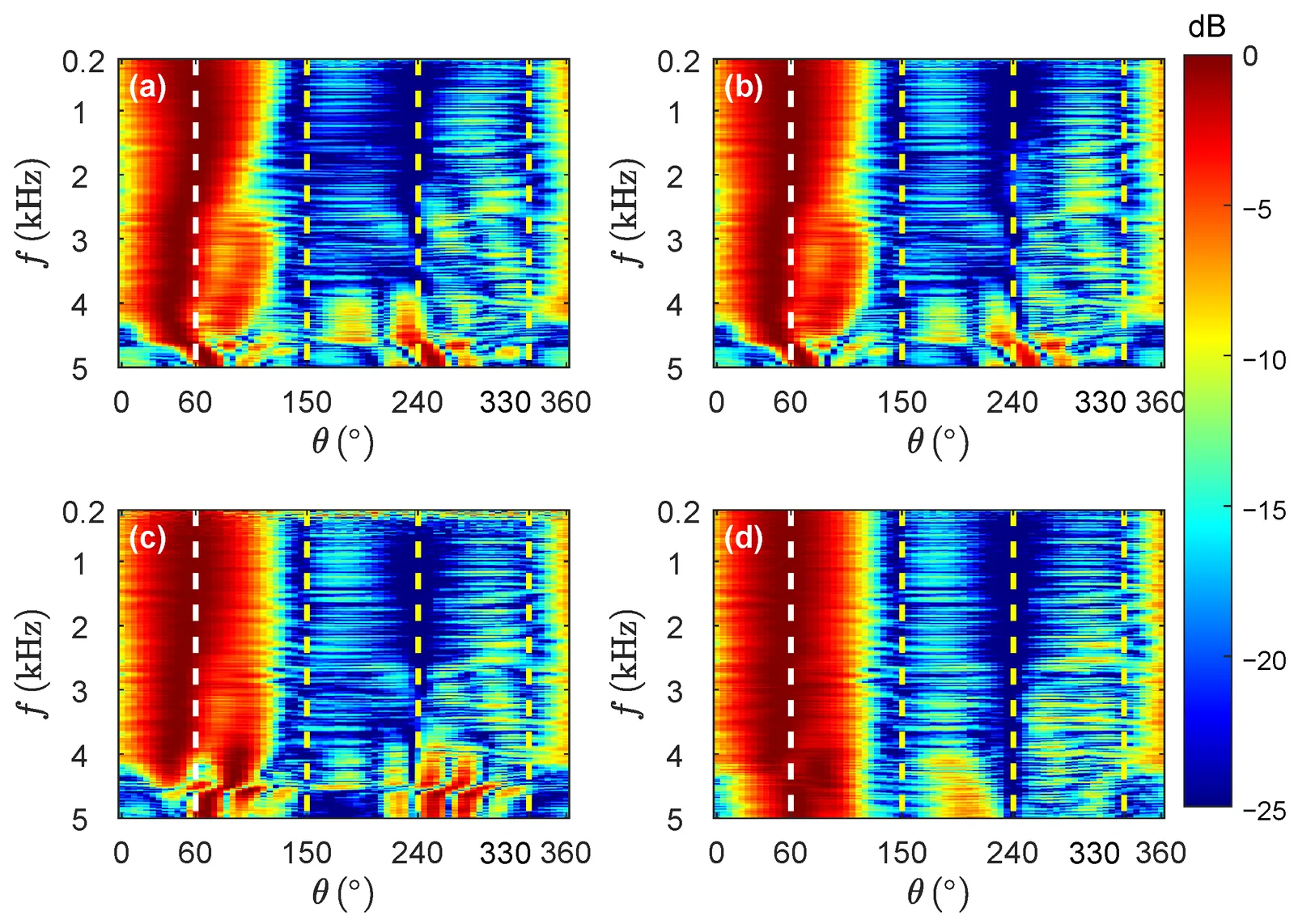
Measured broadband beampattern maps for (**a**) JA with $\left(N_{1},N_{2}\right)=\left(2,\;1\right)$, (**b**) JA with (3, 2), (**c**) JA with (4, 3), and (**d**) INC. White dashed line: $\theta_{s}=60{}^{\circ}$; yellow dashed lines: constrained nulls at 150°, 240°, and 330°.

The same measured data are then used to compare JA [24] and INC under the common target. Figure 13 shows the resulting broadband maps. For the tested truncation orders, JA exhibits frequency-dependent displacement of the main lobe, changes in the sidelobes, and nonuniform null depth. INC maintains closer main-lobe alignment and a more consistent null structure over the measured band. These measured trends are consistent with the numerical comparison in Section 4.5 and support the benefit of the WNG-constrained global-error design for this target and prototype.

To quantify these broadband measurements, Table 3 summarizes four metrics: MSBE, WNG, mean prescribed-null residual, and main-lobe pointing error. Before calculating the MSBE and null residual, each measured response is normalized by its achieved look-direction response. For the experimental results, the MSBE is calculated from the normalized magnitudes of the measured and desired beampatterns, and the repeated 360° endpoint is excluded from the angular average. WNG is calculated from the experimental weight vectors using (16). At each frequency, the residual powers at 150°, 240°, and 330° are averaged before conversion to decibels. The main-lobe pointing error is the absolute angular difference between $\theta_{s}=60{}^{\circ}$ and the measured peak within the expected main-lobe sector, evaluated on the 5° angular grid used in the measurements. The three decibel-valued metrics are averaged over frequency in their respective linear domains before conversion to decibels, whereas the pointing error is averaged in degrees.

For the original experimental configurations summarized in Table 3, INC achieves the lowest band-averaged MSBE of −19.71 dB with a band-averaged WNG of 3.58 dB. Although JA (2, 1) and JA (3, 2) provide higher WNGs, their MSBEs are larger. JA (4, 3) has a slightly lower band-averaged WNG of 3.26 dB but a substantially larger MSBE of −1.40 dB, mainly because of its measured high-frequency deterioration above 4 kHz, as visible in Figure 13c. These results demonstrate that the selected INC configuration provides a favorable balance between robustness and beampattern approximation accuracy. INC also yields the lowest mean prescribed-null residual of −19.89 dB and the smallest mean main-lobe pointing error of 7.5°, indicating closer agreement with the desired beampattern at the prescribed nulls and intended look direction.

**Figure 14:**
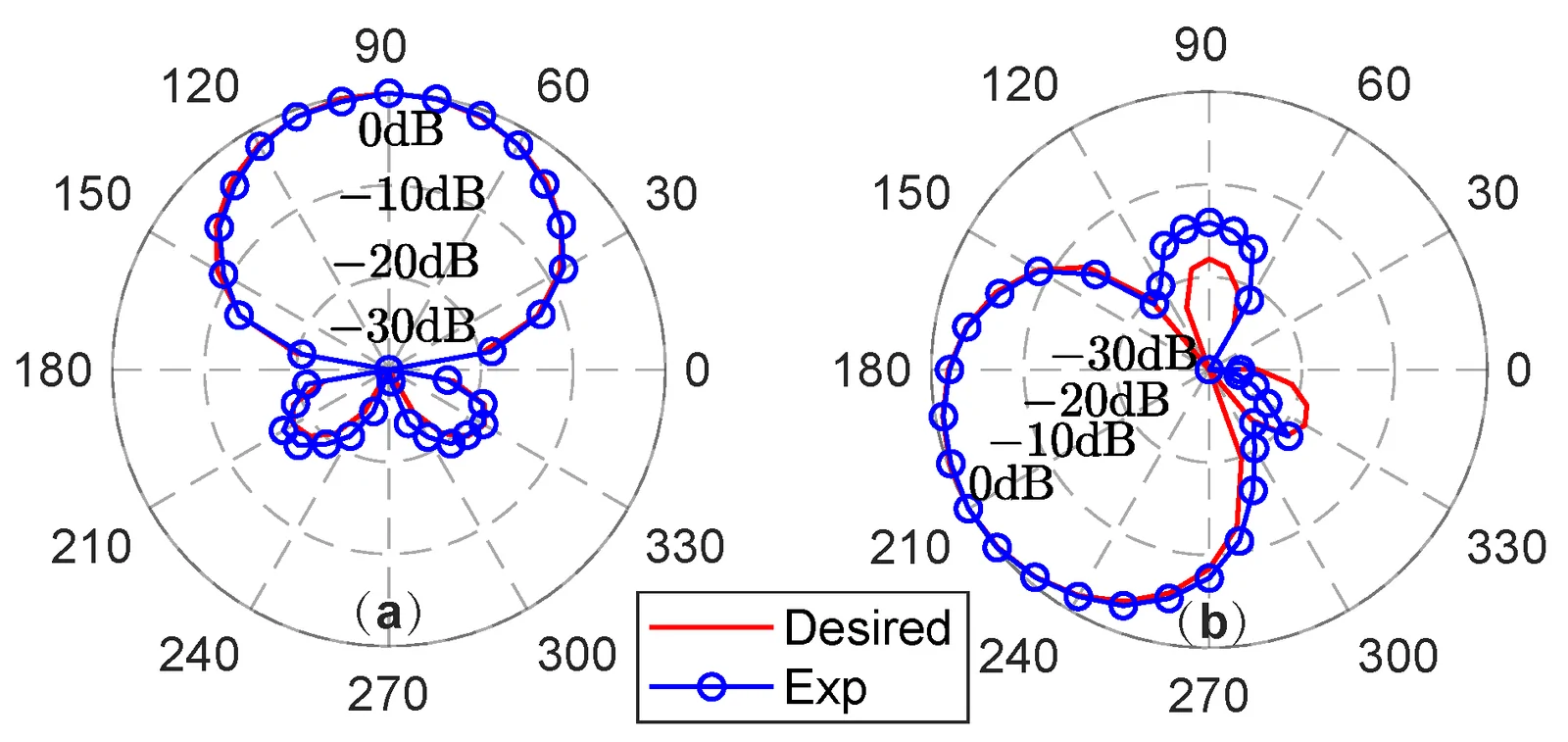
Measured (offline) and desired beampatterns of the second-order differential beamformer at 1 kHz: (**a**) $\theta_{s}=90{}^{\circ}$, (**b**) $\theta_{s}=210{}^{\circ}$.

Finally, Figure 14 compares the measured and reference patterns at 1 kHz for $\theta_{s}=90{}^{\circ}$ and 210°. The peaks follow the two intended directions and the overall responses are preserved, supporting the steering behavior observed in simulation with measured free-field data.

## 6. Conclusions

Building on established null-based target construction and beampattern-error design with WNG control, this work presented their joint realization for a mixed-sensor LSA. A null-derived differential reference is fitted continuously over the array plane, while the look direction and prescribed nulls remain exact affine constraints and a WNG lower bound limits white-noise amplification. Fully steerable operation follows from the existing mixed-sensor LSA manifold; the specific contribution lies in the LSA formulation of this constrained integration and in the analytic evaluation of its continuous-angle MSBE matrices, rather than in a new null-to-target relation, a new WNG metric, or a closed-form solution for the final weights. Numerical results for the evaluated 11-element geometry show lower MSBE than the maximum-WNG NC baseline, a tunable WNG–MSBE trade-off, and stable steering behavior over the tested look directions. Anechoic measurements with the 11-element prototype further show that the main lobe, null structure, and steering behavior are retained in measured free-field responses; this experiment validates the present formulation but is not claimed as the first LSA experiment. The current limitations include higher offline design cost than pseudoinverse-based JA implementations and mismatch sensitivity evaluated only for the representative perturbation ranges considered here. Future work will address rapid coefficient updating, adaptive selection under measured sensor-tolerance distributions, and real-time or reverberant acoustic conditions.

## Figures and Tables

**Table 1:** Comparison with closely related beamforming methods.

Method	Input Specification and Target-Pattern Construction	Null Preservation	Robustness Control	Steering Capability	Tuning Parameters	Applicable Array Type	Experimental Validation
Luo et al. [23]	Ideal directivity pattern +steering direction;predefined analytical target	Approx.	Min.-norm solution; no explicit WNG bound	Full plane	Target order	Omni. + bidirectional LSA	No
Luo et al. [24]	Ideal directivity pattern +steering direction + sensordirectivity;predefined analytical target	Approx.	Min.-norm solution; no explicit WNG bound	Full plane	Two truncation orders; sensor type	Omni. + first-order LSA	Yes
Huang and Habets [27]	Desired steering direction + null positions; no global target pattern (NC)	Exact	Min.-norm solution; no explicit WNG bound	Full plane	No. of nulls; sensor orientations	Mixed-sensor LSA	No
Pan et al. [28]	Order + desired nulls;effective-basesuperposition ($\ell_{1}$/ADMM)	Target level;exact if feasible;otherwiseapproximate	Validityonly; nofilter-levelWNG control	Notaddressedat array level	Order; null set;base set	General DMAtarget-patterntheory	No
Xie et al. [29]	Ideal directivity pattern +fixed steering direction;predefined analytical target	Approx.	Explicit MSBE–WNG trade-off	Fixed endfire	Target order; WNG or MSBE level	Omni. ULA	No
Jin et al. [30]	Fixed desired direction +null positions; no globaltarget pattern (NC)	Exact or relaxed	Quadraticnoise-powerconstraint	Fixed endfire	No. of nulls;tolerance; NCM rank	Omni. ULA	Yes
Jin et al. [31]	Fixed desired direction +SNR-gain specification;no global target pattern	Notaddressed	General SNR-gainconstraints	Fixed endfire	SNR-gain level;covariance matrices	Omni. ULA	Yes
This work	Desired steering direction + prescribed null offsets;null-derived target via [28]	Exact	Global MSBE + explicitWNG lower bound	Full plane	Null set;WNG bound	Omni. + first-orderLSA	Yes

Note: ULA denotes uniform linear array; NC denotes null-constrained; NCM denotes noise covariance matrix.

**Table 2:** Nominal and joint-mismatch performance of INC for different WNG-loss allowances.

*f* (kHz)	*v* (dB)	Achieved WNG (dB)	Nominal MSBE (dB)	Mean MSBE Under Joint Mismatch (dB)
1	5	−5.78	−47.86	−18.81
	10	−10.78	−53.17	−13.81
	20	−14.65	−97.47	−9.95
3	5	5.37	−40.37	−29.14
	10	3.24	−59.23	−27.28
	20	3.24	−59.23	−27.29
5	5	5.28	−39.59	−28.68
	10	4.76	−41.11	−28.27
	20	4.76	−41.11	−28.27

Note: The mean joint-mismatch MSBE is obtained by averaging the linear MSBE over 5000 realizations before conversion to decibels. Lower MSBE indicates a smaller beampattern error.

**Table 3:** Band-averaged quantitative results for the measured broadband responses in Figure 13 over 0.2–5 kHz.

Method	Magnitude MSBE (dB)	WNG (dB)	Mean Null Residual (dB)	Mean Pointing Error (°)
JA (2,1)	−12.91	6.32	−12.73	9.1
JA (3,2)	−15.31	5.71	−16.28	8.2
JA (4,3)	−1.40	3.26	−8.27	12.5
INC, v=10 dB	**−19.71**	3.58	**−19.89**	**7.5**

Note: More negative magnitude MSBE and mean null residual, higher WNG, and smaller mean pointing error indicate better performance; bold values denote the best result for each metric among the compared methods.

## Data Availability

The data presented in this study are available upon reasonable request from the corresponding author.

## Appendix A. Uniqueness of the Null-Implied Reference

This appendix justifies the nonsingularity condition used to reconstruct the reference response from the prescribed null offsets. Let $x_{0}=1$ and $x_{n}=\cos\beta_{n}$ for n=1,…,N. Since $\cos\left(k\beta_{n}\right)=T_{k}\left(x_{n}\right)$, where $T_{k}$ is the *k*th Chebyshev polynomial, (19) can be written as

(A1) $$C_{\beta }=\left[\begin{array}{cccc} T_{0}\left(x_{0}\right) & T_{1}\left(x_{0}\right) & \ldots & T_{N}\left(x_{0}\right)\\ T_{0}\left(x_{1}\right) & T_{1}\left(x_{1}\right) & \ldots & T_{N}\left(x_{1}\right)\\ \vdots & \vdots & \; & \vdots \\ T_{0}\left(x_{N}\right) & T_{1}\left(x_{N}\right) & \ldots & T_{N}\left(x_{N}\right) \end{array}\right].$$

The columns are polynomials of degrees 0, 1, …, N with nonzero leading coefficients. Consequently, $C_{\beta }$ is a generalized Vandermonde matrix and is nonsingular if and only if $x_{0},x_{1},\ldots ,x_{N}$ are pairwise distinct. Equivalently, the reference is unique when $1,\cos\beta_{1},\ldots ,\cos\beta_{N}$ are distinct. Under this condition,

(A2) $$a_{N}=C_{\beta }^{-1}e_{1}.$$

This condition also explains why both members of a symmetric null pair must not be entered as separate rows when reconstructing $a_{N}$: they have the same cosine and therefore provide the same reference constraint.

## Appendix B. Derivation of $\tilde{\Gamma }$, Q, and $\bar{C}$

For the nominal orientation $\phi_{m}=\pi /2$ used in the simulations and prototype, $g_{m}\left(\theta \right)=a_{m}+\left(1-a_{m}\right)\sin\theta$. Substituting (2) into (18) gives

(A3) $$\tilde{\Gamma }=\frac{1}{2\pi }\int_{0}^{2\pi }G\left(\theta \right)d\left(\theta \right)d^{H}\left(\theta \right)G^{H}\left(\theta \right)d\theta .$$

The (m,n)-th element (for m,n=1,2,…,M) is

(A4) $$\begin{array}{cc} {\left[\tilde{\Gamma }\right]}_{m,n} & ={\left[\tilde{\Gamma }\right]}_{m,n}^{I}+{\left[\tilde{\Gamma }\right]}_{m,n}^{\mathrm{II}}+{\left[\tilde{\Gamma }\right]}_{m,n}^{\mathrm{III}},\\ {\left[\tilde{\Gamma }\right]}_{m,n}^{I} & =\frac{a_{m}a_{n}}{2\pi }\int_{0}^{2\pi }e^{jk(x_{m}-x_{n})\cos\theta }d\theta ,\\ {\left[\tilde{\Gamma }\right]}_{m,n}^{\mathrm{II}} & =\frac{\left(a_{m}+a_{n}-2a_{m}a_{n}\right)}{2\pi }\int_{0}^{2\pi }e^{jk(x_{m}-x_{n})\cos\theta }\sin\theta d\theta ,\\ {\left[\tilde{\Gamma }\right]}_{m,n}^{\mathrm{III}} & =\frac{\left(1-a_{m}-a_{n}+a_{m}a_{n}\right)}{2\pi }\int_{0}^{2\pi }e^{jk(x_{m}-x_{n})\cos\theta }\sin^{2}\theta d\theta . \end{array}$$

From the Jacobi–Anger series expansion

(A5) $$e^{jk(x_{m}-x_{n})\cos\theta }=\sum_{n^{\prime }=-\infty }^{+\infty }j^{n^{\prime }}J_{n^{\prime }}\left(kx_{m}-kx_{n}\right)e^{jn^{\prime }\theta },$$

the orthogonality of the circular harmonics

(A6) $$\frac{1}{2\pi }\int_{0}^{2\pi }e^{j\tilde{n}\theta }d\theta =\left\{\begin{array}{cc} 1, & \tilde{n}=0,\\ 0, & \tilde{n}\neq 0, \end{array}\right.$$

and the Euler’s formula,

(A7) $$e^{j\theta }=\cos\theta +j\sin\theta ,$$

we have

(A8) $$\begin{array}{cc} \; & \frac{1}{2\pi }\int_{0}^{2\pi }e^{jk(x_{m}-x_{n})\cos\theta }d\theta =J_{0}\left(kx_{m}-kx_{n}\right),\\ \; & \frac{1}{2\pi }\int_{0}^{2\pi }e^{jk(x_{m}-x_{n})\cos\theta }\sin\theta d\theta =0,\\ \; & \frac{1}{2\pi }\int_{0}^{2\pi }e^{jk(x_{m}-x_{n})\cos\theta }\sin^{2}\theta d\theta =\\ & \frac{1}{2}\left[J_{0}\left(kx_{m}-kx_{n}\right)+J_{2}\left(kx_{m}-kx_{n}\right)\right], \end{array}$$

where $J_{n}\left(\cdot \right)$ is the Bessel function of the first kind of order *n*. Substituting (A8) into (A4), we obtain

(A9) $$\begin{array}{cc} {\left[\tilde{\Gamma }\right]}_{m,n}= & \frac{1}{2}\left[1-\left(a_{m}+a_{n}\right)+3a_{m}a_{n}\right]J_{0}\left(kx_{m}-kx_{n}\right)\\ \; & +\frac{1}{2}\left(1-a_{m}\right)\left(1-a_{n}\right)J_{2}\left(kx_{m}-kx_{n}\right). \end{array}$$

where k≜2πf/c is the wavenumber.

Substituting $\tilde{d}\left(\theta \right)$ and $c_{N,\theta_{s}}\left(\theta \right)$ into (24), the (m,n+1)th entry of Q, for m=1,…,M and n=0,…,N, is

(A10) $$\begin{array}{cc} \; & {\left[Q\right]}_{m,n+1}={\left[Q\right]}_{m,n+1}^{I}+{\left[Q\right]}_{m,n+1}^{\mathrm{II}}+{\left[Q\right]}_{m,n+1}^{\mathrm{III}}+{\left[Q\right]}_{m,n+1}^{\mathrm{IV}},\\ \; & {\left[Q\right]}_{m,n+1}^{I}=\frac{a_{m}\cos\left(n\theta_{s}\right)}{2\pi }\int_{0}^{2\pi }e^{jkx_{m}\cos\theta }\cos\left(n\theta \right)d\theta ,\\ \; & {\left[Q\right]}_{m,n+1}^{\mathrm{II}}=\frac{a_{m}\sin\left(n\theta_{s}\right)}{2\pi }\int_{0}^{2\pi }e^{jkx_{m}\cos\theta }\sin\left(n\theta \right)d\theta ,\\ \; & {\left[Q\right]}_{m,n+1}^{\mathrm{III}}=\frac{\left(1-a_{m}\right)\cos\left(n\theta_{s}\right)}{2\pi }\int_{0}^{2\pi }e^{jkx_{m}\cos\theta }\sin\theta \cos\left(n\theta \right)d\theta ,\\ \; & {\left[Q\right]}_{m,n+1}^{\mathrm{IV}}=\frac{\left(1-a_{m}\right)\sin\left(n\theta_{s}\right)}{2\pi }\int_{0}^{2\pi }e^{jkx_{m}\cos\theta }\sin\theta \sin\left(n\theta \right)d\theta . \end{array}$$

From (A5) and (A7), we have

(A11) $$\begin{array}{cc} \; & \frac{1}{2\pi }\int_{0}^{2\pi }e^{jkx_{m}\cos\theta }\cos\left(n\theta \right)d\theta =j^{n}J_{n}\left(kx_{m}\right),\\ \; & \frac{1}{2\pi }\int_{0}^{2\pi }e^{jkx_{m}\cos\theta }\sin\left(n\theta \right)d\theta =0,\\ \; & \frac{1}{2\pi }\int_{0}^{2\pi }e^{jkx_{m}\cos\theta }\sin\theta \cos\left(n\theta \right)d\theta =0,\\ \; & \frac{1}{2\pi }\int_{0}^{2\pi }e^{jkx_{m}\cos\theta }\sin\theta \sin\left(n\theta \right)d\theta =\\ & -\frac{1}{2}\left[j^{n+1}J_{n+1}\left(kx_{m}\right)-j^{n-1}J_{n-1}\left(kx_{m}\right)\right]. \end{array}$$

By combining (A10) and (A11), we obtain

(A12) $$\begin{array}{cc} {\left[Q\right]}_{m,n+1}= & j^{n}J_{n}\left(kx_{m}\right)a_{m}\cos\left(n\theta_{s}\right)-\frac{1-a_{m}}{2}\sin\left(n\theta_{s}\right)\\ \; & \left[j^{n+1}J_{n+1}\left(kx_{m}\right)-j^{n-1}J_{n-1}\left(kx_{m}\right)\right]. \end{array}$$

Substituting $c_{N,\theta_{s}}\left(\theta \right)$ into (26), the (m,n)th entry of $\bar{C}$, for m,n=1,…,N+1, is

(A13) $${\left[\bar{C}\right]}_{m,n}=\frac{1}{2\pi }\int_{0}^{2\pi }\cos\left[\left(m-1\right)\left(\theta -\theta_{s}\right)\right]\cos\left[\left(n-1\right)\left(\theta -\theta_{s}\right)\right]d\theta .$$

By trigonometric orthogonality, $\bar{C}$ is diagonal, and

(A14) $${\left[\bar{C}\right]}_{m,n}=\left\{\begin{array}{cc} 1, & m=n=1,\\ 1/2, & m=n>1,\\ 0, & m\neq n, \end{array}\right.$$
